# The Brazilian-adapted Working Alliance Inventory: preliminary report on the psychometric properties of the original and short revised versions

**DOI:** 10.1590/2237-6089-2019-0099

**Published:** 2020-10-08

**Authors:** Fernanda Barcellos Serralta, Silvia Pereira da Cruz Benetti, Pricilla Braga Laskoski, Daniel Abs

**Affiliations:** 1 Programa de Pós-Graduação em Psicologia Universidade do Vale do Rio dos Sinos São LeopoldoRS Brazil Programa de Pós-Graduação em Psicologia, Universidade do Vale do Rio dos Sinos (UNISINOS), São Leopoldo, RS, Brazil.; 2 Hospital de Clínicas de Porto Alegre Porto AlegreRS Brazil Hospital de Clínicas de Porto Alegre (HCPA), Porto Alegre, RS, Brazil.; 3 Programa de Pós-Graduação em Psiquiatria e Ciências do Comportamento Universidade Federal do Rio Grande do Sul Porto AlegreRS Brazil Programa de Pós-Graduação em Psiquiatria e Ciências do Comportamento, Universidade Federal do Rio Grande do Sul (UFRGS), Porto Alegre, RS, Brazil.; 4 UFRGS Porto AlegreRS Brazil UFRGS, Porto Alegre, RS, Brazil.

**Keywords:** Therapeutic alliance, psychometric properties, reliability, validity, Working Alliance Inventory

## Abstract

**Introduction:**

Alliance is an essential component of all psychotherapies and a consistent predictor of its outcomes. The Working Alliance Inventory (WAI) is a widely used and psychometrically sound measure of alliance. It assesses three key aspects of the construct: a) agreement on the tasks of therapy; b) agreement on the therapeutic goals; and c) development of an affective bond.

**Objective:**

To preliminarily analyze the psychometric properties of the Brazilian version of both the original, 36-item WAI, and of the short form revised, 16-item version (WAI-SR).

**Methods:**

The sample comprised 201 psychodynamic psychotherapy patients. Alliance assessments were made after the 4th treatment session.

**Results:**

The inventory adapted to Brazilian Portuguese, in both the original and short forms, appears to be reliable and valid to measure alliance and its dimensions by clients in psychotherapy. Further studies are needed to replicate and expand the findings.

## Introduction

The patient-therapist relationship is a factor common to all psychotherapeutic processes. Relationship factors are at least as much relevant as the particular psychotherapy approach adopted to predict treatment outcome.^1^ Therapeutic alliance (working alliance or simply alliance) is by far the most widely researched common factor in the context of psychotherapy research worldwide.^2^ A recent meta-analysis of 295 independent studies (published between 1978 and 2017) that covered more than 30,000 patients for face-to-face and Internet-based psychotherapy confirmed the robustness of the positive relation between alliance and outcome. These data remain consistent across raters (clients, therapists, observers, and others, such as family, group members or staff), alliance and outcome measures, treatment approaches, patient characteristics, and countries. The study findings indicated a trend towards slightly lower observer-rated effects when compared with client-rated effects in terms of alliance-outcome correlation (whereas therapist and other categories did not differ from client-rated alliance).^3^ Thus, measuring alliance is fundamental for conducting process-outcome research of any kind, as well as for clinical assessments in routine practice.

It is noteworthy that the diversity of instruments designed to measure alliance reflects the variety of co-existing constructs.^4^ The synthesis of Horvath et al. identified the existence of dozens of alliance scales.^5^ However, two-thirds of the studies used one of the four best-known alliance measures, namely: the California Psychotherapy Alliance Scale (CALPAS),^6^ the Helping Alliance Questionnaire (HAq),^7^ the Vanderbilt Psychotherapy Process Scale (VPPS),^8^ and the Working Alliance Inventory (WAI).^9 , 10^ Among these, the WAI is the most widely used and thus will be the focus of our interest.

The WAI is based on Bordin’s pan-theoretical tripartite model of working alliance.^11^ This model conceives alliance as a collaborative relationship built by three components: the bond, the agreement upon therapeutic goals, and the agreement upon therapy tasks. Different therapies can differ in terms of which of these components are more emphasized. In addition, since alliance is a dyadic construct, and therefore built by interpersonal interactions, patient and therapist might experience it differently. Most of the empirical base of the construct is sustained in research considering the patient’s view of alliance.^5^

Despite the notorious development of alliance studies worldwide, Brazilian clinicians and researchers do not have many options when it comes to choosing a valid measure of alliance. We conducted a review on the main national databases (Index Psi and LILACS) to identify alliance assessment instruments available in Brazilian Portuguese. We found limited studies, with few instruments: the California Psychotherapy Alliance Scale – Patient Version (CALPAS-P), the Helping Alliance Questionnaire (HAq II), and the WAI.^12^ The Brazilian version of the CALPAS-P has shown limited to acceptable reliability, with Cronbach’s alphas of subscales varying from 0.56 to 0.84 and a total scale score of 0.90.^13^ The Brazilian versions of HAq II (patient and therapist) were used to explore the relationship between alliance and defense mechanism. Psychometric properties of these versions are not reported.^14^ As for the WAI, a theoretical paper by Prado & Meyer mentioned an adaptation conducted by Paulo Machado e Cristiano Nabuco de Abreu from the Portuguese version of it.^15^ However, the adaptation study has not been published, and the psychometric characteristics of this version are unknown.

The WAI has three main forms: therapist (WAI-T), client (WAI-C), and observer (WAI-O). Other variants of the inventory have also been developed, e.g., the 12-item Working Alliance Inventory – Short (WAI-S)^16^ and its revised version (WAI-SR).^17^ A 6-item version of the instrument was developed for use repeatedly over treatment allowing to measure alliance change on a session-to-session basis.^18^ The original inventory has 36 items, 12 for each subscale: emotional bond between patient and therapist; agreement of goals; and tasks. Several studies confirmed the three factor-structure of the scale, but also noted a high correlation between the goal and task subscales.^10 , 19 , 20^ Other authors^21 , 22^ prefer a two-factor model that combines the goal and task subscales.

As for the development of the first short version (WAI-S), Tracey & Kokotovic^16^ selected the four highest loading items on each of the three WAI dimensions based on confirmatory factor analysis (CFA), and then performed a second CFA. Although the model presented a good fit to the data and the measure became popular, methodological limitations of the study were raised. To address them, Hatcher & Gillaspy^17^ reanalyzed the factor structure of the WAI-S. The results did not confirm the good fit of the model. The authors then developed the instrument’s short form revised (WAI-SR). First, they performed a principal axis factor analysis of WAI and then a direct oblimin rotation. This analysis yielded six factors. As negatively worded items formed separate factors from the positively worded ones, they decided to focus on the three factors with positively worded items. The three positive factors corresponded closely to the original WAI dimensions. For the first factor (goal), the items selected consisted of three original goal items and one original task item. Three original task items and one original goal item comprised factor two (task). The third factor (bond) was formed by four original bond items. The final 12-item WAI-SR was tested in two samples. The model with three correlated factors showed a superior fit compared with both the one-factor and two-factor models.

Munder et al.^20^ investigated and compared the psychometric properties of the WAI-SR in German outpatients (n = 88) and inpatients (n = 243). Their results were widely in accordance with those of Hatcher & Gillaspy,^17^ supporting the three-factor model. In other words, the findings suggested that the WAI-SR is able to distinguish the task and goal aspects of the therapeutic alliance. Conversely, Falkenström et al.^23^ used Bayesian structural equation modeling with zero mean and small variance prior to testing the factor structure of the WAI-SR in three different samples (one American and two Swedish; n = 235, 634, and 234, respectively). They found a high intercorrelation between the task and goal factors in the three-factor model across all three samples, indicating that in general these factors cannot be differentiated. However, they considered it premature to rule out this model in favor of the two-factor model (combined task and goal scales) and called for studies using subsamples of specific patient groups or treatment orientations.

Recently, with the authorization of the copyright holder, our research group developed an adaptation of the WAI to Brazilian Portuguese. The translation-back translation procedures were conducted by independent bilingual experts following guidelines for cross-cultural adaptation for self-reported measures.^24 , 25^ In this paper, we address the last step of this process, i.e., the examination of the psychometric properties of the instrument. The study aims to help enhance confidence in the WAI versions available in the Brazilian context by examining their reliability and evidencing construct validity. As suggested by other authors,^20 , 23^ we tested the original Bordin’s three-factor model in a specific setting (i.e., a sample of patients undergoing psychoanalytic psychotherapy). Since we chose to study the WAI client version, like most previous studies,^3^ our discussion will privilege patient’s perspective of the alliance.

## Method

### Participants and setting

The sample comprised 201 patients receiving treatment at a psychoanalytic psychotherapy outpatient service and is derived from a study on relationships between personality, bond and change processes in psychoanalytic/psychodynamic psychotherapy. The research ethics committee from Universidade do Vale do Rio dos Sinos approved the study protocol (CAAE: 39120214.6.0000.5344). In accordance with ethical standards, prior to filling out the instruments, patients received a free and informed consent form which included information about the objectives of the study and their right to freely collaborate or decline with no interference with treatment, as well as of publication of the results while preserving confidentiality. The profile of the participants has been described in detail previously.^26^ The sample included 139 female and 62 male patients with age ranging from 18 and 67 years (mean = 32.48, standard deviation = 12.35). Most of the participants had university education (69.1%) and presented neurotic symptoms like anxiety, depression, and interpersonal problems.

### Measure: Working Alliance Inventory

The WAI^9^ comprises 36 items rated on a 7-point Likert-type scale. Fourteen items are negatively worded and must be reversed when computing scores. The scale has three subscales, based on Bordin’s working alliance model: bond, goal and task. The short form revised (WAI-SR)^17^ comprises 12 items of the original scale, 4 in each subscale.

### Procedures

Data were collected in the context of the broader research protocol from which this study is derived. Informed consent to voluntary participation in the study was obtained before treatment, during screening, by a research assistant. Patients answered two other measures prior to therapy. Alliance assessments were made after the 4th treatment session. Patients and their respective therapists received an enclosed envelope containing a series of self-report instruments, including the WAI, to be returned in the next session.

### Statistical analysis

In addition to descriptive analysis, with results expressed as means and standard deviations, CFA^27 - 29^ was applied to verify the factorial structure of the scales in the sample, thus providing evidence of construct validity of both versions of the WAI (the 36-item original version and also the 12-item short form revised). These analyses were performed using the AMOS software version 20.

CFA comprises a set of strategies to assess the factorial structure of a given instrument using a variance-covariance matrix in comparison to the data collected.^29^ For this analysis, parameters are previously set based on the literature, and later estimated. The maximum likelihood method was used to estimate the parameters. Adjustment indicators were checked to understand how much the estimated model fit the data collected. Many authors^27 - 29^ recommend the use of the root mean square error of approximation (RMSEA), the chi-square, the non-normed fit index (NNFI), and the comparative fit index (CFI) in CFA analysis. In an ideal setting, NNFI and CFI should be greater than 0.900, and desirably greater than 0.950, and RMSEA should be less than 0.080, not exceeding 0.100. Confirmatory factorial models with good adjustment indexes suggest that the factorial structure estimated is reasonable for the data collected. Internal consistency analyses based on Cronbach’s alphas and composite reliability^30^ were also performed.

## Results

In order to examine whether the original Bordin’s theoretical model of working alliance fit the clients’ responses to the WAI, CFAs were conducted on the theorized three correlated factors in both WAI and WAI-SR. Table 1 summarizes these results.

**Table 1:** - Goodness of fit in different confirmatory factor analysis models for the WAI and the WAI-SR

Model	χ^2^	df	p	NNFI	CFI	RMSEA (90%)
WAI						
WAI Bond	74.02	46	0.005	0.924	0.947	0.056 (0.031-0.079)
WAI Task	65.81	48	0.045	0.972	0.98	0.044 (0.007-0.068)
WAI Goal	63.13	45	0.038	0.961	0.973	0.045 (0.011-0.070)
WAI Total	1,095.18	564	< 0.001	0.808	0.828	0.069 (0.063-0.075)
WAI-SR						
WAI-SR Bond	1.129	2	0.569	1	1	0.000 (0.000-0.121)
WAI-SR Task	3.15	2	0.206	0.988	0.996	0.055 (0.000-0.164)
WAI-SR Goal	0.347	2	0.841	1	1	0.000 (0.000-0.081)
WAI-SR total	80.79	47	0.002	0.955	0.968	0.061 (0.038-0.084)

CFI = comparative fit index; df = degrees of freedom; NNFI = non-normed fit index; RMSEA = root mean square error of approximation; WAI = Working Alliance Inventory; WAI-SR = Working Alliance Inventory – Short Form Revised.

Considering multiple parameters (NNFI, CFI, RMSEA and chi-square), both WAI and WAI-SR showed acceptable fit, with WAI-SR showing a better fit than the 36-item original scale.

The scales’ Cronbach’s alphas are presented in Table 2 .

**Table 2:** - Coefficient alphas for the WAI and the WAI-SR subscales and total scores

Dimension	WAI alpha	WAI-SR alpha
Bond	0.794	0.651
Task	0.882	0.838
Goal	0.828	0.862
Total	0.935	0.884

WAI = Working Alliance Inventory; WAI-SR = Working Alliance Inventory – Short Form Revised.

As expected, correlations between the subscales and the WAI total alliance score were all strong and significant (≤ 0.001): r = 0.86 (bond), r = 0.94 (task) and r = 0.95 (goal). A similar correlation pattern was found between the WAI-SR bond, task and goal subscales and the WAI-SR total score: r = 0.65, r = 0.87, and r = 0.84, respectively. Correlations between the subscales and the total score of the two versions of the measure were also estimated ( Table 3 ).

**Table 3:** Comparisons of correlations between subscales and total scores of the WAI and the WAI-SR

Scale or subscale pair comparisons	r
Bond-Task	
WAI vs. WAI-SR	0.663*
WAI-SR vs. WAI	0.463*
WAI vs. WAI	0.890*
WAI-SR vs. WAI-SR	0.492*
Bond-Goal	
WAI vs. WAI-SR	0.664*
WAI-SR vs. WAI	0.531*
WAI vs. WAI	0.721*
WAI-SR vs. WAI-SR	0.497*
Task-Goal	
WAI vs. WAI-SR	0.802*
WAI-SR vs. WAI	0.807*
WAI vs. WAI	0.890*
WAI-SR vs. WAI-SR	0.807*
Bond- Bond	
WAI vs. WAI-SR	0.835*
Task-Task	
WAI vs. WAI-SR	0.879*
Goal-Goal	
WAI vs. WAI-SR	0.850*

WAI = Working Alliance Inventory; WAI-SR = Working Alliance Inventory – Short Form Revised.

* p ≤ 0.001.

As can be observed in Table 3 , all WAI and WAI-SR subscales were highly correlated. Therefore, both the WAI and the WAI-SR were considered suitable to measure alliance and its components in a very similar way.

## Discussion

The process of cross-cultural adaptation of a measure intends to produce equivalence between the original and the adapted versions based on item content. A well-done cultural adaptation process, however, does not assure retention of psychometric properties such as validity and reliability. Thus, it is necessary to test the psychometric properties of the adapted measure as well.^24^

Bordin^11^ conceived that alliance is developed and sustained by an ongoing negotiation between patient and therapist, comprising three facets: agreement on goals, collaboration on tasks, and bond. The WAI was built based on this model.^10^ However, some controversy exists about whether the WAI behaves according to this model, or whether a two-factor model combining the task and goals scales should be favored.^22^

After a carefully designed step-by-step adaptation process of the WAI to Brazilian Portuguese, we reported a preliminary analysis of psychometric properties of two versions of the measure (the original 36-item inventory and a short revised version, with 12 items) applied to a sample of psychoanalytic psychotherapy patients based on the original three factor model.

We found that both forms of measure are reliable, with total score alphas of 0.88 for WAI-SR and 0.93 for WAI. All subscale score alphas were good (> 0.80), except for the bond scale, which showed an acceptable (nearly good) coefficient for the WAI (0.79) and a questionable (nearly acceptable) coefficient for the WAI-SR (0.65).

In one of the original studies of the WAI, the bond scale also showed a questionable alpha.^10^ In other studies, however, all WAI scales showed good reliability.^17^ Therefore, the lower homogeneity found in bond scale items in our sample could be an artifact due to characteristics of our sample. For example, based on Bordin’s ideas, it has been hypothesized that alliance may be developed differently in different therapies. This assumption remains little explored in research.^31^ Bond, in comparison with task and goal factors, is more subjective. Its perception by psychodynamic therapy patients at early stages of treatment could present more variations, perhaps as a result of the ambivalence and persecutory anxiety provoked by the psychodynamic setting and techniques. Considering the reliability indexes found for bond scales, we recommend further studies. Until then, to clinicians and researchers using the Brazilian version of the WAI here assessed, we recommend caution when interpreting results derived from the application of this scale alone.

Since the number of items generally affects the coefficient (i.e., more items tend to inflate the estimate), we consider both versions equivalent in terms of internal consistency. Moreover, once a high alpha value (> 0.90) may suggest that the measure has redundancies and could benefit from reduction,^30^ the WAI-SR apparently might be favored.

Construct validity was examined by CFA procedures that showed that both the WAI and the WAI-SR presented adequate fit to the tripartite model of alliance proposed by Bordin. In this model, alliance is conceived as a collaborative relationship between patient and therapist that can be understood by three combined facets that define the quality of the relationship: the affective bond, and the mutual agreement upon therapy goals and tasks. The concept of bond refers to a positive attachment between patient and therapist, including love, trust, and acceptance. The goals are the target of the intervention, i.e., the expected outcome. Tasks refer to behaviors, activities and cognitions that are performed in therapy. They are the essence of the therapeutic process and ideally might be perceived as meaningful and efficacious.

There is excellent quality in both versions of the scale. Because the WAI has more items and more parameters, it is more likely to have a higher chi-square, which affects other indices. Thus, comparatively, we considered that the WAI-SR performed better, not so much because its CFI indicators were higher – this was expected due to the smaller number of items –, but mainly because of the set of adjustment indicators found, the correlations obtained and the parsimony of having fewer items to compose its measurement structure. It is important to mention that, given the high CFI values found for both WAI-SR bond and WAI-SR goal scales, additional analyses were performed with the lavaan of R package, using maximum likelihood (ML) and weighted least square mean and variance adjusted (WLSMV), and similar results were obtained for CFI, chi-square, and RMSEA, thus excluding any hypothesis of specification problems for these values. Therefore, we conclude that the two scales presented good adjustment to the three-factor alliance model. Any conclusion about the best version of the WAI to be used for alliance measurement is premature.

The study has many limitations. It is a preliminary study conducted on a sample limited to a single psychotherapy institution. Alliance was measured at a single (initial) stage of treatment. Perception of the alliance is known to be influenced by several factors, including treatment stage. Therefore, it is important to consider that subsequent studies on inventory properties should examine alliance in multiple contexts and treatment phases. Although further investigations of the Brazilian versions of both WAI and WAI-SR are needed, the present study contributes by providing Brazilian researchers and therapists with a description of the psychometric properties of two versions of a sound inventory designed to measure alliance that apparently maintained its properties after translation into Brazilian Portuguese.

## Conclusion

This is the first study on psychometric properties of the Brazilian-adapted client version of the WAI. The inventory, in both original and short forms, appears to be reliable and valid to measure alliance and its dimensions by clients. Therefore, Brazilian clinicians and researchers can use either the WAI or the WAI-SR to assess client alliance. Results should be replicated in other samples and contexts. In addition, other psychometric qualities (e.g., predictive validity) should be explored further.
